# Critical roles of phosphorylation in multiple signaling pathways during the progression of osteoarthritis

**DOI:** 10.1016/j.gendis.2026.102182

**Published:** 2026-04-06

**Authors:** Jingwen Li, Muhammad Umar, Xin Dai, Yu-Ching Hsu, Hongxun Sang, Di Chen, Ke Lu

**Affiliations:** a Department of Biomedical Engineering, Southern University of Science and Technology, Shenzhen, Guangdong 518055, China; b Faculty of Pharmaceutical Sciences, Shenzhen University of Advanced Technology, Shenzhen, Guangdong 518107, China; c Department of Orthopaedics, Shenzhen Hospital, Southern Medical University, Shenzhen, Guangdong 518101, China

**Keywords:** Osteoarthritis, Phosphorylation, Post-translational modifications, Protein phosphatases, Signal transduction

## Abstract

Osteoarthritis (OA) is a progressive degenerative joint disease characterized by synovial inflammation, cartilage degradation and alterations of subchondral bone remodeling. The complex pathogenesis of OA is driven by the dysregulation of multiple intracellular signaling pathways. Among the various post-translational modifications (PTMs) that regulate protein levels and functions, reversible protein phosphorylation serves as a critical “molecular switch” in the pathophysiology of OA. In this review, we comprehensively summarized the current status of how phosphorylation and dephosphorylation dynamics within key signaling pathways, including MAPK, PI3K/AKT, and NF-κB pathways, govern the progression of OA. We focus on how site-specific phosphorylation activates or inhibits these pathways, thereby controlling chondrocyte metabolism, apoptosis, and inflammatory responses. Furthermore, we discuss the therapeutic potential of small molecules targeting these phosphorylation events and provide a perspective on future research directions, such as the unexplored role of protein phosphatases, the application of high-throughput phosphoproteomics, and the integration of artificial intelligence for novel target discovery. A deeper understanding of the phosphorylation network in OA is essential for providing the basis for the development of more precise and effective therapeutic strategies.

## Introduction

Post-translational modifications (PTMs) are covalent chemical alterations to proteins after their synthesis, a process that dramatically expands the functional diversity of the proteome, thereby orchestrating almost all cellular processes, including signal transduction, protein localization, enzyme activity, and protein–protein interaction.^1^ While the human genome contains approximately 20,000–25,000 genes, the proteome comprises over a million distinct proteins, with PTMs being the primary mechanism responsible for this exponential increase in complexity. These modifications include the covalent addition of chemical functional groups (e.g., phosphate, acetyl), complex molecules (e.g., sugars, lipids), or even entire proteins (e.g., ubiquitin and SUMO), as well as the proteolytic cleavage of peptide bonds. These reactions are typically catalyzed by specific enzymes, such as kinases, phosphatases, transferases, and ligases, and can be either reversible or irreversible, allowing for both dynamic regulation and permanent functional changes.

In physiological and pathological conditions, the chondrocyte phenotype is orchestrated by a complex network of PTMs. The pathogenesis of OA is rooted in a profound imbalance of these signaling networks, leading to a metabolic shift in chondrocytes from an anabolic to a catabolic state, which culminates in the progressive degradation of the extracellular matrix (ECM) and loss of joint function. A growing body of evidence demonstrates that various PTMs are intimately involved in OA pathogenesis. For instance, O-GlcNAc modification levels are found to be increased in OA cartilage, inducing hypertrophic differentiation in chondrocytes.^2^ Ubiquitination can accelerate or alleviate OA progression by targeting specific proteins for degradation.^3^ Lactylation, a newly identified PTM, may play a critical role in rheumatoid arthritis (RA), a related joint disease.^4^ Deamidation of proteins in the chondrocyte extracellular matrix suggests differential joint turnover in OA.^5^ Lysine malonylation, regulated by Sirt5, is associated with chondrocyte metabolism and OA development.^6^ While this review focuses on phosphorylation, acknowledging the involvement of other PTMs is vital, as they likely interact with phosphorylation pathways, influencing OA progression. Biological systems are inherently complex, and PTMs do not operate in isolation; understanding their interplay is crucial for a comprehensive grasp of OA pathogenesis (Fig. 1).

**Figure 1:**
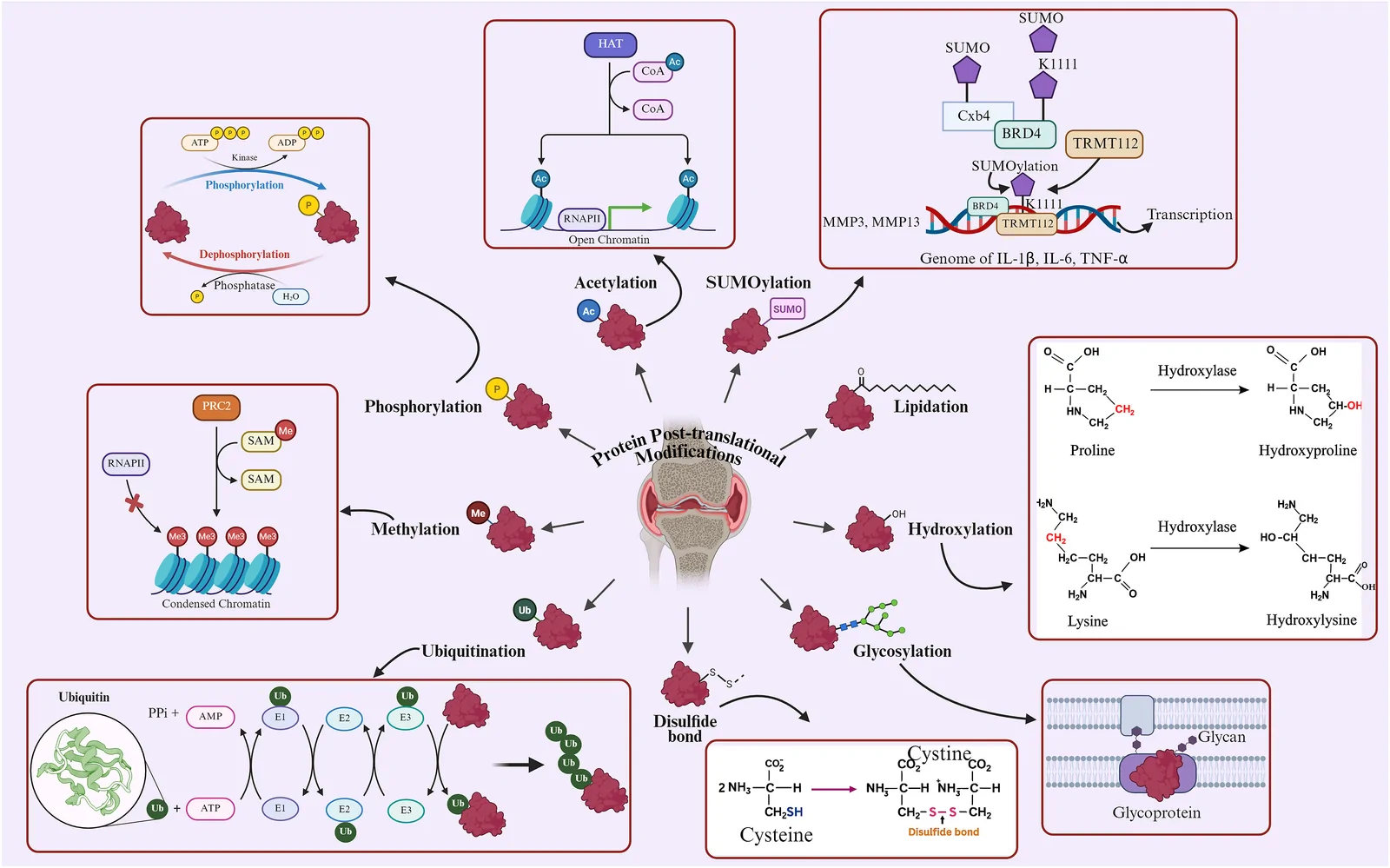
Overview of post-translational modifications (PTMs) of proteins involved in OA. Chemical modifications such as phosphorylation (P), acetylation (Ac), methylation (Me), and redox-based modifications (S–S) are reversible. Polypeptide modifications, including ubiquitination (Ub) and SUMOylation (SUMO), are also enzymatically reversible. Additionally, complex molecule modifications such as hydroxylation, glycosylation and lipidation are reversible processes contributing to protein regulation.

Among the myriad of known PTMs, reversible protein phosphorylation stands out as one of the most ubiquitous and influential regulatory mechanisms. This dynamic process of phosphorylation and dephosphorylation operates as a sophisticated “molecular switch”, enabling rapid and reversible control over protein function. The state of this switch is tightly controlled by two opposing families of enzymes: protein kinases, which catalyze the transfer of a phosphate group from ATP to a specific serine, threonine, or tyrosine residue on substrate proteins, and protein phosphatases, which remove these phosphate groups through hydrolysis.^7^ The precise balance between kinase and phosphatase activity is fundamental to maintaining cellular homeostasis and ensuring proper physiological responses.

In recent years, a large body of evidence has established that phosphorylation is a central regulatory hub in OA pathology. Extensive research has highlighted its critical role across multiple pathways. For instance, the phosphorylation-dependent activation of the NF-κB pathway is a key driver of pro-inflammatory gene expression in chondrocytes.^8^ In OA, IL-1β induces phosphorylation of the YAP protein at the Ser 127 site, leading to cartilage degeneration. The bone resorption process in osteoporosis (OP) is driven by phosphorylation of the NF-κB pathway, and 18β-glycyrrhetinic acid can alleviate bone loss by inhibiting I-κB phosphorylation. Dysregulated phosphorylation within the PI3K/AKT pathway profoundly impacts chondrocyte survival and apoptosis.^9^ The canonical wingless/integrated (Wnt)/β-catenin signaling pathway could play a unique role in the pathogenesis of arthritis,^10^ while protein phosphorylation controls Wnt/β-catenin signaling.^11^

Despite these significant individual findings, the knowledge remains fragmented. A comprehensive synthesis that integrates these diverse phosphorylation events into a cohesive network view of OA pathogenesis is currently lacking. This review, therefore, aims to systematically collate and analyze the roles of phosphorylation modifications within key signaling pathways in OA. By doing so, we aim to provide a deeper understanding of the disease's molecular underpinnings and highlight the value of targeting phosphorylation/dephosphorylation for therapeutic intervention, thereby justifying the rationale and importance of this work.

## Molecular signaling involved in the course of OA

The destructive communication between the synovium and cartilage is orchestrated at the molecular level by intracellular signaling pathways, with phosphorylation acting as the master conductor.^12^ A closer examination of the signaling networks in OA reveals a complex interplay between the synovium and cartilage, with phosphorylation acting as the core mechanism driving this pathological dialogue. In synovial cells, phosphorylation is the primary engine of the inflammatory component of OA. Synovitis, which is present even in early OA, contributes significantly to pain and disease progression.^13^ This inflammation is driven by phosphorylation-dependent signaling cascades within fibroblast-like synoviocytes (FLSs). The mitogen-activated protein kinase (MAPK) pathways, including ERK, JNK, and p38, are intensely studied in this context. The pro-inflammatory cytokines, like TNF-α and IL-1β, activate these kinase cascades and phosphorylate downstream targets that promote the production of inflammatory mediators, such as IL-6 and prostaglandin E2 (PGE2), as well as matrix metalloproteinases (MMPs). The importance of phosphorylation in synovial inflammation is underscored by research in rheumatoid arthritis (RA), a related inflammatory joint disease, and elevated levels of phosphorylated PDGFRα/β in synovial cells suggest involvement in synovial cell accumulation and inflammation,^14^ where the successful clinical use of JAK inhibitors like tofacitinib directly demonstrates the therapeutic potential of targeting these phosphorylation-dependent pathways.^13^ In a mouse model of RA, TNF-α increases inflammatory factor expression in synovial fibroblasts via the Toll-like receptor 3-mediated ERK/AKT signaling pathway (phosphorylation-dependent).^15^ This suggests that targeting kinases within the synovium could be a viable strategy to reduce the joint inflammation that fuels OA. Beyond the synovium, the subchondral bone represents another critical region of the joint affected by phosphorylation-dependent pathology that actively communicates with cartilage. Unlike its protective role in cartilage, high levels of active TGF-β in OA subchondral bone induce the excessive phosphorylation of Smad 2/3 in mesenchymal progenitor cells.^16^ This uncouples bone remodeling and promotes the formation of osteoid islets and aberrant angiogenesis, which subsequently invade the overlying cartilage. Furthermore, the phosphorylation-dependent regulation of Wnt/β-catenin signaling in osteocytes is tightly linked to subchondral sclerosis, a hallmark of OA that alters the mechanical environment of the joint.^17^ Thus, OA progression is driven by a synchronized ‘tripartite’ signaling network involving the cartilage, synovium, and subchondral bone, rather than by chondrocytes in isolation. Here, we will introduce the pathways that play a crucial role in the course of OA, as well as the regulatory effects of related drugs on the targets in these pathways.

### MAPK signaling pathway

The mitogen-activated protein kinase (MAPK) pathway is a conserved cascade central to cellular responses to various extracellular stimuli like inflammatory cytokines, growth factors, and stress.^12^ It typically involves three kinases: MAPKKK, MAPKK, and MAPK.^12^ In OA, inflammatory cytokines (e.g., IL-1β, TNF-α) can activate MAPKKKs, which phosphorylate and activate MAPKKs (MKKs). MKKs then phosphorylate and activate MAPK family members, including ERK, JNK, and p38 MAPK.^12^ Activated MAPKs subsequently phosphorylate downstream targets, including transcription factors (e.g., AP-1, NF-κB) and protein kinases, thereby regulating the gene expression involved in inflammation, cartilage catabolism, and apoptosis. In osteocytes, growth factors, such as TGF-β, activate fibronectin and collagen expression through membrane integrins, activate the FAK/Pyk2-MAPK pathway, or sense mechanical stimuli through primary cilia to regulate the nuclear translocation of YAP/TAZ, converting mechanical signals into biochemical signals and modulating the balance between osteogenesis and osteoclastogenesis. Abnormal mechanical signal transduction in the subchondral bone is the core mechanism of OA induced by abnormal mechanical loading. For example, JNK phosphorylation activates AP-1, leading to enhanced collagenase gene expression, potentially contributing to excessive ECM degradation in RA. p38 MAPK phosphorylation is associated with chondrocyte apoptosis and pro-inflammatory factor production in OA.^18^ In chondrocytes, TAK1, as a core molecule at the intersection of the NF-κB and MAPK pathways, undergoes K63 ubiquitination mediated by TRAF2/6 upon stimulation by IL-1β, TNF-α, TGF-β, and mechanical loading. This process induces conformational changes and exposes the kinase active site, allowing it to bind to TAB2/3 to form an active complex. Simultaneously, it phosphorylates and activates key nodes in the NF-κB pathway, such as the IKK complex and MKKs. Additionally, it can connect with TGF-β, Wnt, PI3K/Akt and other pathways by phosphorylating Smad 7, FoxO 1 and other molecules, thereby regulating chondrocyte autophagy, phenotype maintenance and matrix metabolism.^19^ TAK1 can also directly phosphorylate the Ser 204 site of Smad 3, regulating its transcriptional activity and affecting the synthesis of the chondrocyte matrix. In OA, inflammatory factors activate TAK1, phosphorylate Smad7, promote its binding to TGF-βRII, inhibit the phosphorylation of Smad 2/3, and block the chondroprotective effect of TGF-β, forming a pathological loop of inflammation → TAK1 activation → TGF-β signaling inhibition → cartilage degeneration.^20^ The ERK pathway is also implicated in OA cartilage destruction.^21^ MAPKs (ERK1/2, JNK, p38) are key kinases activated by phosphorylation. Inhibitors targeting specific MAPKs exist, such as SB203580 (inhibitor of p38) and SP600125 (inhibitor of JNK) (Fig. 2).^22^

**Figure 2:**
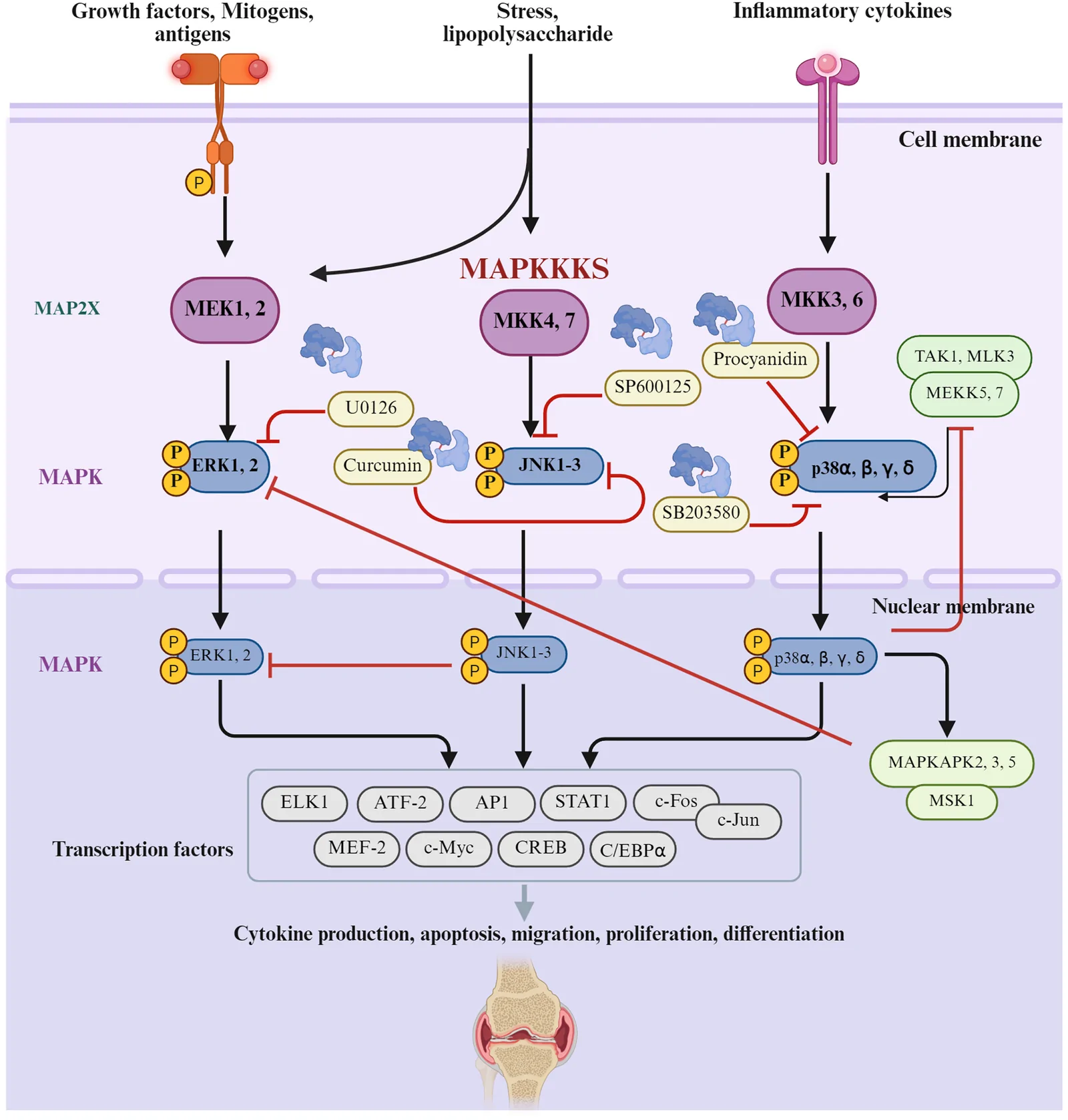
MAPK signaling pathway (ERK, JNK, p38).

### SP600125

SP600125 is a selective JNK inhibitor that acts by blocking the JNK signaling pathway. It has been shown to inhibit the phosphorylation of c-Jun in a dose-dependent manner (IC50 of 5–10 μM). SP600125 directly inhibits JNK-mediated c-Jun phosphorylation and blocks downstream pro-inflammatory and pro-apoptotic signaling.^23^ In osteoblasts, SP600125 affects osteogenesis-related genes by inhibiting JNK phosphorylation and completely blocking CBP-mediated ATF4 expression. The phosphorylation of p38 and ATF2 was partially inhibited at high concentration (50 μM), but not at low concentration (25 μM). In TNF-α-induced bone marrow mesenchymal stromal cells (BMSCs), SP600125 restored the chondrogenic capacity and alleviated the inhibition of chondrogenic differentiation by TNF-α through inhibiting the JNK pathway.^23^ In an *in vitro* ear cartilage regeneration model, SP600125 significantly reduced the expression of TNF-α, IL-1β and MMP13, and inhibited extracellular matrix (ECM) degradation. By blocking JNK signaling, SP600125 reduced chondrocyte apoptosis and the inflammatory response and delayed the progression of OA. Studies have shown that SP600125 reduces cartilage destruction and improves joint function by inhibiting the JNK pathway in a mouse model of OA. A high concentration of SP600125 (50 μM) may produce off-target effects on non-JNK targets (e.g., p38), and the dose should be carefully controlled.

### SB203580

SB203580 is a classical p38 MAPK inhibitor, but the study found a non-specific effect on the regulation of NF-κB activity, which may be related to the ERK pathway. p38 MAPK selective inhibition: SB203580 is a pyridinylimidazole analog, which inhibits the catalytic activity of p38 MAPK by competitively binding to its ATP-binding site. SB203580 blocks the activation of p38 downstream substrates such as MAPKAPK-2/3, thereby inhibiting the phosphorylation of transcription factors such as HSP27 and ATF-2, and ultimately reducing the expression of inflammatory factors (e.g., IL-1β, TNF-α, and IL-6).^24^ In RAW264.7 macrophages, SB203580 significantly inhibited LPS-induced TNF-α and IL-6 mRNA transcription.^25^ SB203580 inhibited the expression of pro-inflammatory factors, such as IL-1β and TNF-α, by blocking the p38 MAPK pathway, thereby reducing synovial inflammation. In a synovitis model, SB203580 inhibited the downstream effects of p38 phosphorylation upon TLR4 activation and reduced IL-1β release, suggesting its potential application in OA treatment.^26^ As a classical p38 MAPK inhibitor, SB203580 inhibits inflammatory responses by selectively blocking kinase activity, but its nonspecific effects (e.g., ERK/JNK feedback activation) need to be carefully evaluated. In OA models, it exerts anti-inflammatory effects through down-regulation of factors such as IL-1β, providing a rationale for targeting p38 therapies.

### U0126

U0126 significantly reduced Thr202/Tyr204 phosphorylation of ERK1/2 by blocking MEK1/2 activity, inhibited the activation of downstream transcription factors (e.g., c-Fos, c-Jun), and reduced the expression of pro-inflammatory factors (IL-6, TNF-α) and matrix-degrading enzymes (MMP13, ADAMTS-5).^27^ U0126 is a selective MEK inhibitor that inhibits downstream ERK phosphorylation (p-ERK) by blocking MEK1/2 activity. U0126 significantly reduces the protein expression of MMP3 in OA cartilage by inhibiting ERK phosphorylation, thereby slowing cartilage degeneration.^27^ Similar to U0126, the inhibitor SP600125 or SB203580, also reduces MMP3 expression and activates autophagy by inhibiting their respective targets (phosphorylation of JNK and p38).

### NF-κB signaling pathway

The NF-κB pathway plays a critical role in inflammatory and immune responses.^28^ In the resting state, NF-κB transcription factors are bound to inhibitory I-κB proteins in the cytoplasm.^28^ Pro-inflammatory stimuli (e.g., IL-1β, TNF-α) activate the I-κB kinase (IKK) complex.^28^ IKK phosphorylates I-κB proteins, triggering their ubiquitination and proteasomal degradation.^28^ NF-κB then translocates to the nucleus, binds to promoter regions of target genes, and induces the transcription of pro-inflammatory cytokines, chemokines, and metalloproteinases.^28^ The phosphorylation status of NF-κB is crucial for its nuclear translocation and transcriptional activity.^28^ In OA, activation of the NF-κB pathway contributes to joint inflammation and cartilage destruction.^28^ The NF-κB pathway was the strongest pro-inflammatory pathway in FLS. Inflammatory factors such as TNF-α, IL-1β, and LPS activate the IKK complex, leading to the degradation of I-κB and the release of NF-κB p65/*p*50 into the nucleus, which transcriptionally regulates pro-inflammatory/invasive factors such as IL-6, IL-8, and MMPs. Sustained activation of this pathway in RA results in the synovial hyperplasia. In OA, FLSs are stimulated by IL-6/IL-1β, activating the JAK1/2-STAT3 pathway. Phosphorylated STAT3 enters the nucleus and forms a complex with NF-κB p65, jointly regulating the transcription of IL-6, IL-8, and MMP3. Secreted IL-6 then activates the JAK/STAT pathway through autocrine/paracrine mechanisms, forming a positive feedback loop of IL-6 → JAK/STAT → NF-κB → IL-6 and maintaining low-grade synovial inflammation. The inflammatory factors generated through this cross-talk diffuse through synovial fluid, becoming important exogenous triggers for the activation of pathways in cartilage and subchondral bone (Fig. 3).

**Figure 3:**
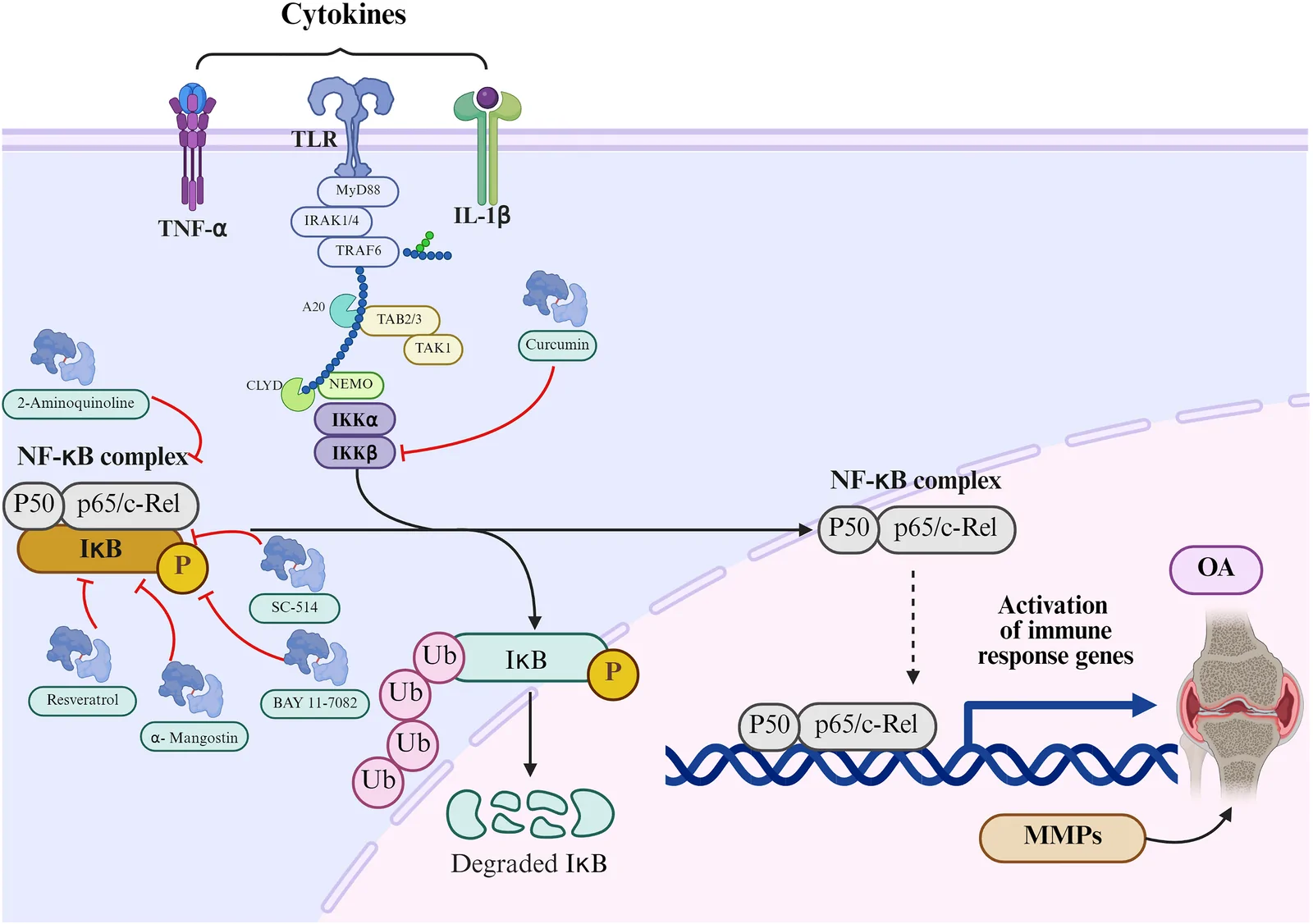
NF-κB signaling pathway.

### BAY-11-7082

BAY-11-7082 indirectly inhibits NF-κB activation by inhibiting I-κB phosphorylation and degradation. Experimental data show that BAY-11-7082 significantly reversed the inhibition of alkaline phosphatase (ALP) expression by TNF-α and partially restored the differentiation ability of osteoblasts by blocking NF-κB activation. This suggests that BAY-11–7082 may ameliorate bone metabolism abnormalities by regulating the NF-κB pathway, which is one of the important pathological mechanisms of OA.^29^ BAY-11-7082 was found to inhibit the TNF-α-induced NF-κB signaling pathway, thereby reducing the expression of inducible nitric oxide synthase (iNOS). Although the study focused on rheumatoid arthritis (RA)-associated bone loss, the NF-κB pathway also plays a key role in cartilage degeneration and inflammatory responses in OA.^30^ The study also found that BAY-11-7082 significantly reduced visfatin (a pro-inflammatory adipokine)-induced chondrocyte apoptosis and oxidative stress by inhibiting the NF-κB pathway. Specifically, pre-treatment with BAY-11-7082 decreased the expression of miR-34a and miR-181a, while restoring the levels of antioxidant enzymes (e.g., SOD-2, CAT) and the anti-apoptotic protein BCL2. These findings suggest the potential therapeutic value of BAY-11-7082 in protecting chondrocytes from inflammatory injury.^31^

### Curcumin

Curcumin inhibits the phosphorylation of I-κBα by blocking the activity of the IKK complex, preventing its degradation and nuclear translocation of NF-κB.^32^ In B cells, curcumin inhibits the phosphorylation of ERK, p38, and I-κB, which further reduces the activation of NF-κB. Inhibition of STAT3 phosphorylation. Through inhibition of STAT3 phosphorylation, curcumin exerts its antiproliferative effects in cancer.^33^^,^^34^ Curcumin further protects joints by regulating the Wnt/β-catenin pathway and inhibiting TNF-α expression.^35^

### Rapamycin

Rapamycin significantly activates chondrocyte autophagy and reduces NF-κB signaling activation caused by inflammatory stimuli (e.g., IL-1β). This is manifested by a reduction in nuclear translocation of phosphorylated P65, which reduces the transcriptional activity of NF-κB. I-κBα protein degradation is inhibited, maintaining the binding of NF-κB to its inhibitory proteins and preventing its entry into the nucleus.^36^ Rapamycin inhibits the expression of matrix-degrading enzymes such as MMP3, MMP9, and Adamts5 through the above mechanisms, thereby attenuating cartilage destruction. Rapamycin also affects the β-catenin pathway through inhibition of β-catenin signaling and attenuates articular cartilage degeneration in an OA mouse model.^37^ Direct topical joint application of rapamycin needs to be optimized due to its low solubility and rapid clearance from the body. The core mechanism of rapamycin is to protect chondrocytes by inhibiting the NF-κB pathway through activation of autophagy, reducing inflammation-related P65 phosphorylation and catabolic gene expression. Other pathways (e.g., β-catenin) and cross-regulation (e.g., AMPK) may complement its action. Future studies are needed to optimize the dosing strategy to enhance its clinical efficacy.

### NGF/TrkA signaling pathway

Nerve growth factor (NGF) is a neurotrophic factor originally recognized for its role in neuronal survival and differentiation. However, in recent years, studies have shown that NGF plays a crucial role in chronic pain states, particularly in OA. The expression levels of both NGF and its high-affinity receptor tropomyosin receptor kinase A (TrkA) are significantly elevated in the joint fluid, synovium, cartilage and sensory nerves of OA patients. Treatments targeting NGF or TrkA (e.g., the anti-NGF monoclonal antibody Tanezumab) have shown significant OA analgesic effects in clinical trials, further confirming the centrality of the NGF signaling pathway in OA pain mechanisms (Fig. 4). And phosphorylation plays a central role in the regulation of the downstream effects of the NGF signaling pathway. NGF acts primarily by binding to its high-affinity tyrosine kinase receptor TrkA. When NGF binds to its cell surface TrkA receptor, it induces dimerization of the TrkA receptor, leading to the activation of the intracellular domain of TrkA, which in turn undergoes autophosphorylation of specific tyrosine residues within the receptor molecule. This is the first and most critical phosphorylation event in the initiation of NGF signaling.^38^^,^^39^ The phosphorylated TrkA receptor serves as a “mooring platform” capable of recruiting and binding to a variety of intracellular signaling proteins and junction proteins containing SH2 (Src homology 2) or PTB (phosphotyrosine binding) domains, such as Shc, FRS2 (fibroblast growth factor receptor) and PLCγ (phospholipase C-gamma). Recruitment and/or phosphorylation of these proteins by TrkA initiates several downstream signaling cascades, the most prominent of which are the MAPK pathway and the PI3K/AKT pathway. Recruited junctional proteins (e.g., Shc, FRS2) activate the small *G* protein Ras. Activated Ras initiates a kinase phosphorylation cascade: Raf → MEK → ERK (extracellular signal-regulated kinase). In this process, Raf phosphorylates and activates MEK, which in turn phosphorylates and activates ERK. Activated ERK (phosphorylated ERK) can enter the nucleus and alter gene expression (e.g., increase the expression of nociceptive-related ion channels, neuropeptides) by phosphorylating regulatory transcription factors (e.g., CREB, c-Fos) or phosphorylate other target proteins in the cytoplasm and directly regulate their functions.^29^, ^30^, ^31^, ^32^, ^33^, ^34^, ^35^, ^36^, ^37^, ^38^, ^39^ The p38 MAPK pathway can also be activated by the NGF/TrkA and PI3K/AKT pathways (phosphatidylinositol 3-kinase/protein kinase B). Phosphorylated TrkA recruits and activates PI3K, which catalyzes the production of the lipid second messenger PIP3, which recruits AKT (also known as PKB) and PDK1 to the cell membrane. The p38 MAPK pathway is also activated by NGF/TrkA, which participates in the modulation of inflammation. AKT is then fully activated by phosphorylation by kinases such as PDK1 and mTORC2. Activated AKT (phosphorylated AKT) can phosphorylate a variety of downstream targets that regulate cell survival, proliferation, metabolism, and inflammatory responses.^39^^,^^40^

**Figure 4:**
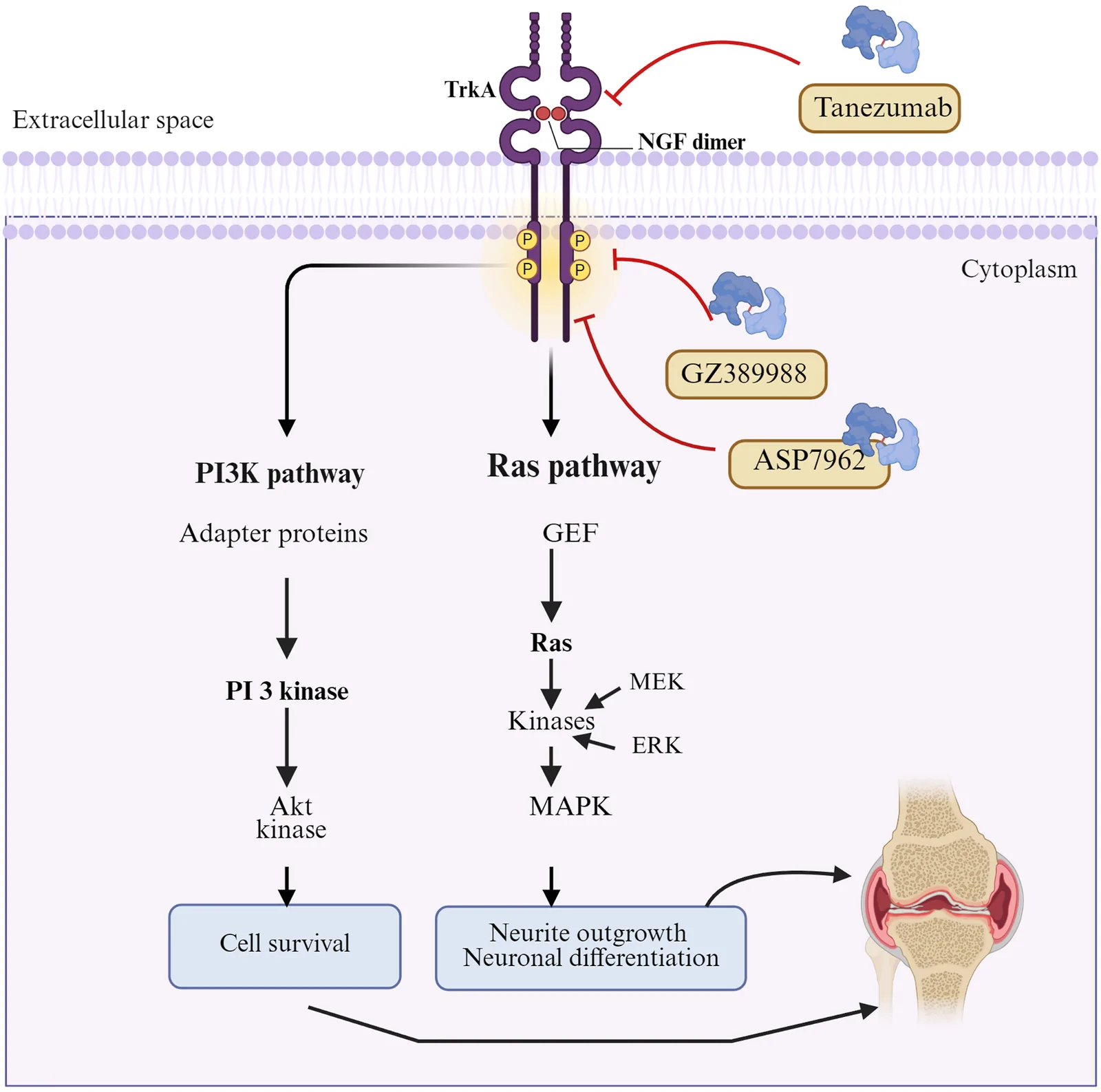
NGF/TrkA signaling pathway.

### ASP7962 and GZ389988

TrkA is the high-affinity receptor for NGF, which plays a significant role in OA pain generation and nervous system sensitization. NGF binding induces TrkA autophosphorylation, activating downstream signaling pathways that lead to pain signal transmission and inflammatory mediator release. TrkA inhibitors (e.g., ASP7962, GZ389988) block NGF binding or inhibit the kinase activity of TrkA and preventing TrkA phosphorylation and subsequent downstream signaling activation, thereby alleviating OA-related pain. ASP7962 specifically inhibits ATP-induced substrate phosphorylation by human TrkA.^41^ GZ389988 also acts as a TrkA inhibitor, likely involving inhibition of TrkA phosphorylation.^42^

### Tanezumab

NGF is a neurotrophin and pro-inflammatory cytokine that is crucial in OA pain pathogenesis. Tanezumab is a monoclonal antibody that binds to and neutralizes NGF, preventing it from binding to its receptor, TrkA. Since NGF is the upstream signal for TrkA phosphorylation and activation, tanezumab indirectly inhibits TrkA phosphorylation and downstream pain signaling by reducing NGF availability.^43^

### PI3K/AKT/mTOR signaling pathway

The PI3K/AKT/mTOR pathway is an intracellular signaling route regulating cell growth, proliferation, survival, and metabolism.^44^ Phosphatidylinositol 3-kinase (PI3K) is activated by phosphorylating phosphatidylinositol (4,5)-bisphosphate (PIP2) to generate phosphatidylinositol (3,4,5)-trisphosphate (PIP3).^44^ PIP3 recruits and activates the protein kinase AKT, which then phosphorylates and regulates numerous downstream targets, including the mammalian target of rapamycin (mTOR).^44^ mTOR is a serine/threonine kinase, containing two distinct complexes, mTORC1 and mTORC2, which regulate processes like protein synthesis, cell growth, and autophagy. In OA, this pathway is involved in the synovial inflammation, subchondral bone dysfunction and cartilage degradation. Under pathological conditions, pro-inflammatory FLSs undergo phenotypic reprogramming, and the signaling pathways shift toward pro-inflammatory, invasive, and anti-apoptotic directions. It cross-links with the NF-κB pathway. TNF-α and IL-1β synergistically regulate the secretion of inflammatory factors, MMP expression, and cell proliferation of FLS by activating the MAPK pathway; the ERK pathway is also involved in the migration and invasion of FLS, and the p38 pathway regulates the post-transcriptional modification of inflammatory factors.^45^ For instance, inhibition of PI3K/AKT/mTOR phosphorylation has a protective role in cartilage integrity (Fig. 5). The cross-talk between the IKK complex and the PI3K/Akt/mTOR and Wnt/β-catenin pathways respectively constitutes the core mechanisms underlying cell metabolic reprogramming, anti-apoptosis, chondrocyte phenotype, and the regulation of bone metabolism in OA: IKK-α phosphorylates the Rictor subunit of mTORC2 to activate the PI3K/Akt pathway.^19^

**Figure 5:**
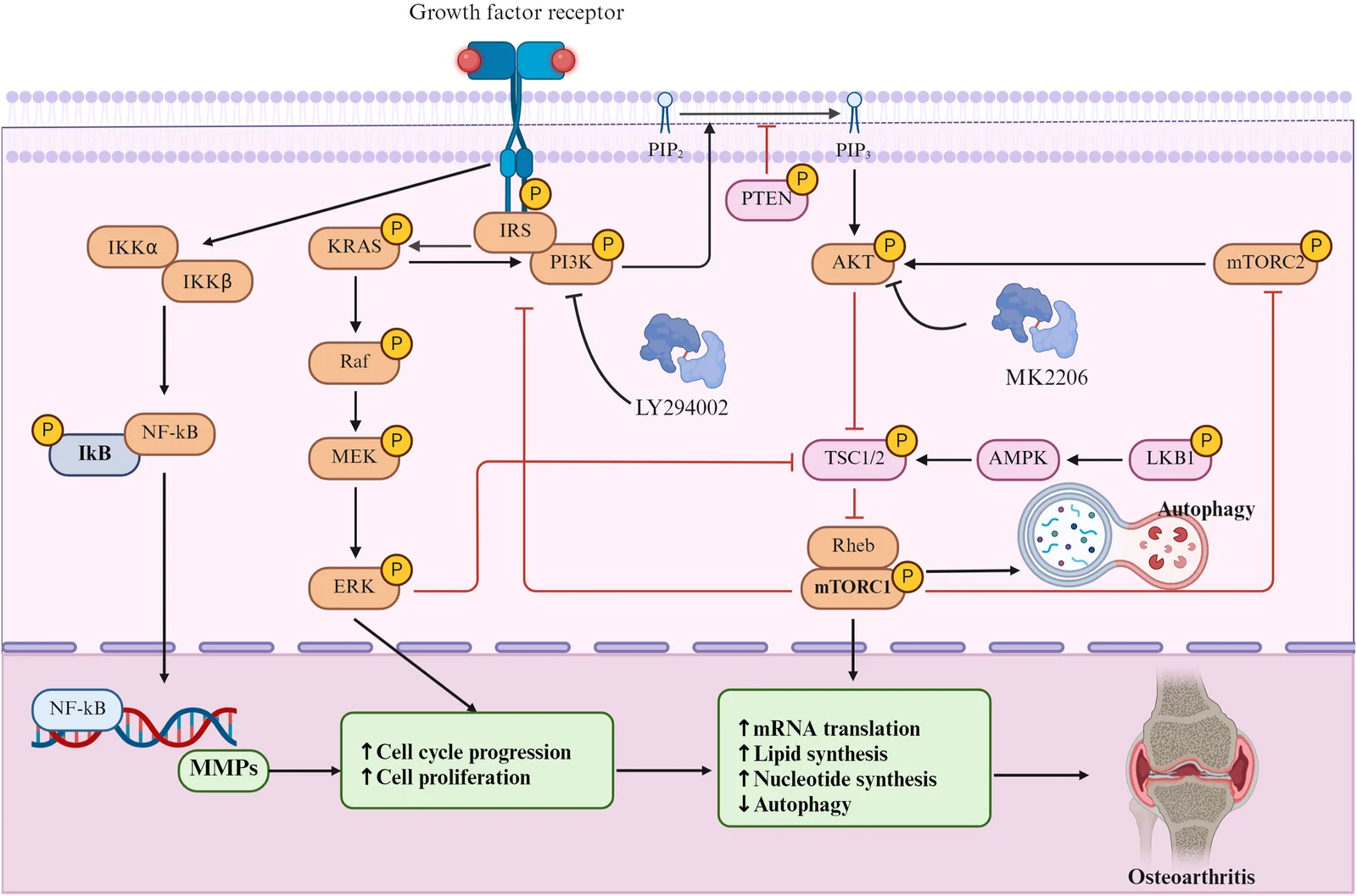
PI3K/AKT/mTOR signaling pathway.

### MK2206

MK2206 blocks the activity of AKT by directly inhibiting its phosphorylation. For example, under static and low shear stress (LSS) conditions, MK2206 significantly inhibited the phosphorylation of AKT at Ser 473 and Thr308 sites, thereby reducing the expression of the inflammatory factor ICAM-1.^46^ In bone marrow mesenchymal stromal cells (BMSCs), MK2206 inhibited IGF-1-induced phosphorylation of AKT, reversed cell proliferation and promoted apoptosis. MK2206 reduced the phosphorylation of FOXO3a by inhibiting AKT activity, thereby restoring its pro-apoptotic function. For example, MK2206 enhanced glucocorticoid-induced apoptosis in osteotriol combination therapy. In chemoresistance models, MK2206 blocked ERK activation and drug resistance by inhibiting AKT phosphorylation at Ser 256 of FOXO1.^47^ MK2206 modulates epigenetic modifications (e.g., H3K27me3) by inhibiting AKT activity, reducing phosphorylation of EZH2, and increasing its methylation and stability.^48^

### LY294002

LY294002 blocks PI3K activity by competitively binding to the ATP site of PI3K, thereby inhibiting the phosphorylation of its downstream target AKT, which is required for activation at two key sites, Ser 473 and Thr308.^49^ For example, in a chronic compression injury (CCI) model, LY294002 significantly blocked AKT phosphorylation at Ser473 and Thr308 sites and alleviated mechanical allodynia. In osteosarcoma cells, LY294002 inhibited AKT phosphorylation in a dose- and time-dependent manner, induced apoptosis and inhibited proliferation.^49^ In a bone cancer pain model, LY294002 reduced the production of reactive oxygen species (ROS) in mitochondria and inflammatory cytokines (e.g., IL-1β, TNF-α) by inhibiting the PI3K/AKT/mTOR pathway, thereby alleviating pain. LY294002 inhibited AKT, mTOR, GSK-3β, and other phosphorylation of key proteins, which in turn regulated processes such as the inflammatory response, oxidative stress, apoptosis and osteogenic differentiation. Together, these mechanisms may attenuate the pathological progression of OA, including cartilage degeneration, osteophyte formation and pain.^49^

### SMAD2/3 signaling pathway

Smad 2 and Smad 3 are key intracellular transcription factors in the transforming growth factor β (TGF-β) signaling pathway.^50^ Upon TGF-β binding to its receptor, receptor kinases are activated, phosphorylating the C-terminus of Smad2 and Smad3.^50^ Phosphorylated Smad2/3 forms a complex with the cofactor Smad 4 and translocates to the nucleus to regulate the transcription of target genes involved in cell growth, differentiation, and extracellular matrix (ECM) production. Phosphorylation sites within the Smad2/3 linker region (e.g., Ser 213 and Ser 204 on Smad3) are crucial for TGF-β signaling and are affected by inflammation in OA.^50^ Inflammation can block the protective effect of TGF-β on chondrocyte hypertrophy via dephosphorylation of the Smad2/3 linker region. Phosphorylation of CKIP-1 regulates the Smad2/3 pathway and influences the chondrocyte phenotype.^51^ Moreover, specific protein phosphatases play pivotal roles in the precise regulation of SMAD signaling dynamics. For instance, small C-terminal domain phosphatases (SCPs) specifically dephosphorylate the inhibitory phosphorylation sites within the linker regions of SMAD2/3 (e.g., Ser 245, Ser 250, and Ser 255),^52^ thereby potentially restoring their transcriptional activity of SMAD2/3. Conversely, protein phosphatase Mg2+/Mn2+-dependent 1 A (PPM1A) functions as a primary negative regulator by directly dephosphorylating the C-terminal SXS motif of SMAD2/3 to terminate TGF-β signaling; notably,^53^ recent studies have demonstrated that inhibition of PPM1A effectively attenuates cartilage degeneration in OA. The Smad and NF-κB/IKK pathways are the core of the intersection. The p65 activated by IKKβ can bind to Smad2/3 to inhibit its nuclear translocation and can also competitively bind to SOX9 to block the chondromatrix synthesis function of the Smad pathway. Meanwhile, Smad3 can reversely bind to p65 to inhibit the pro-inflammatory transcriptional activity of NF-κB. In OA, the imbalance of this negative feedback leads to the obstruction of cartilage repair and the acceleration of matrix degradation. The Smad and Wnt/β-catenin pathways have dual antagonistic/synergistic effects.^54^ Smad3 directly interacts with β-catenin to inhibit its nuclear translocation, while BMP/Smad 1/5/8 and the Wnt pathway synergistically regulate the osteogenic differentiation of subchondral bone. In OA, the excessive activation of the Wnt pathway can block the bone metabolic balance and the function of Smad, leading to bone sclerosis/bone spur formation. Smad also intersects with the MAPK/TAK1 and PI3K/Akt/mTOR pathways. The p38 activated by TAK1 can phosphorylate Smad2/3 to regulate its activity, and Akt can affect the function of bone repair of the BMP pathway by phosphorylating Smad1/5/8. Meanwhile, the Smad pathway can inhibit the pro-metabolic reprogramming effect of PI3K/Akt. In OA, the disorder of this intersection leads to enhanced glycolysis and the persistence of the anti-apoptotic phenotype in cells.^55^

### JAK/STAT signaling pathway

The JAK/STAT pathway transduces extracellular signals (e.g., cytokines, growth factors) to the nucleus to regulate gene expression.^56^ It involves the association and activation of Janus kinases (JAKs) upon receptor binding. JAKs phosphorylate both the receptors and signal transducers and activators of transcription (STAT) proteins.^57^ Phosphorylated STATs dimerize, translocate to the nucleus, bind to DNA, and regulate the transcription of target genes involved in inflammation, immunity, and cell proliferation STAT3 phosphorylation is up-regulated in OA cartilage and associated with inflammation. JAK inhibitors suppress this pathway by blocking the phosphorylation activity of JAKs, thereby reducing inflammatory responses (Fig. 6).^57^

**Figure 6:**
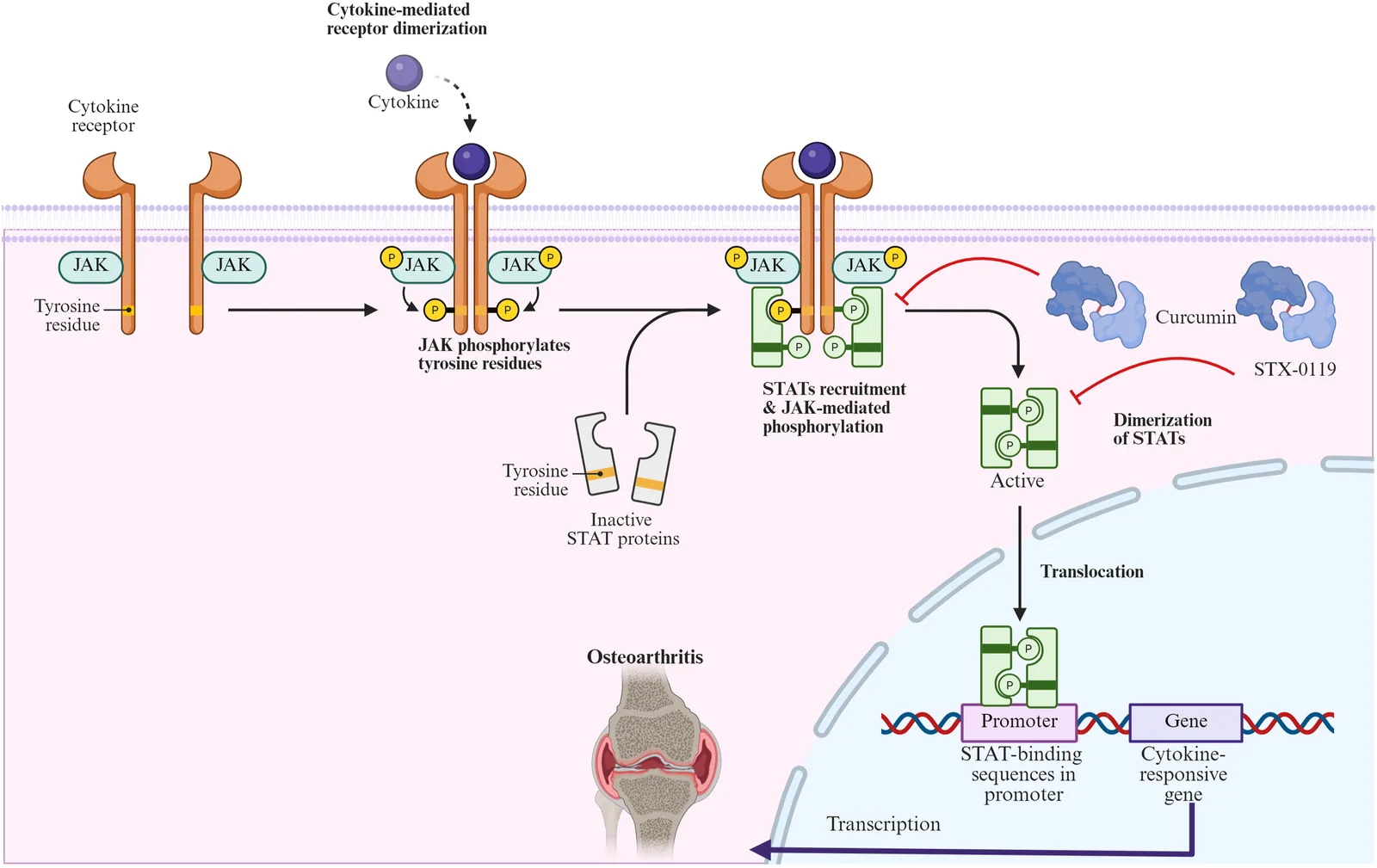
JAK/STAT signaling pathway.

### STX-0119

STX-0119 is a selective inhibitor of STAT3.^56^ STAT3 is a transcription factor playing a key role in mediating inflammatory responses, with elevated phosphorylation levels observed in OA cartilage. STAT3 activation depends on phosphorylation of its tyrosine residues, typically mediated by JAK kinases. Phosphorylated STAT3 dimerizes and translocates to the nucleus, regulating the expression of target genes, including those involved in inflammation and catabolism.^58^ STX-0119 specifically inhibits STAT3 phosphorylation, thereby suppressing inflammatory responses and promoting chondrocyte anabolism, and alleviating OA progression.

### Tofacitinib

Tofacitinib is a broad-spectrum JAK inhibitor, with particularly strong effects on the inhibition of JAK1 and JAK3.^59^ In OA research, tofacitinib has been found to protect the extracellular matrix of chondrocytes by inhibiting the JAK1/STAT3 signaling pathway. The specific mechanism may involve up-regulating chondrocyte autophagy, thereby reducing cartilage degradation.^59^ Clinically, tofacitinib has been approved for the treatment of RA, which provides a theoretical basis for its application in OA, as it can effectively inhibit the inflammatory response mediated by the JAK/STAT pathway. A study established an OA animal model and observed the protective effect of tofacitinib on OA cartilage, further emphasizing the key role of the JAK1/STAT3 pathway in OA.^56^

Above, we have summarized the influence and research of molecular signaling pathways in the course of OA, and have screened and sorted the currently reported research targets. The recognition of phosphorylation event as a central node in OA pathogenesis has opened new avenues for therapeutic intervention. Significant progress has been made in identifying the specific kinases that drive destructive signaling cascades, positioning them as highly attractive targets for the development of the first true DMOADs. The rationale is compelling: inhibiting a key upstream kinase could theoretically block multiple downstream catabolic and inflammatory events, offering a powerful strategy to halt disease progression.

In conclusion, the paradigm shift toward a molecular understanding centered on phosphorylation has revolutionized the field and provided a rational basis for targeted drug design. The intricate nature of these signaling pathways and the difficulties in translating preclinical findings into safe and effective therapies underscore the urgent need for continued, in-depth research. A more granular understanding of the specific kinases, phosphatases, and their complex interplay within the joint is an essential prerequisite for finally developing therapies that can modify the course of this debilitating disease.

The primary focus on kinase cascades often obscures the fundamental role of phosphorylation in keeping a reversible, dynamic equilibrium. In OA, the pathogenesis involves not only the hyperactivation of 'writers' (kinases) but also the critical failure of 'erasers' (phosphatases). Dual-specificity phosphatases (DUSPs), particularly DUSP1 (MKP-1),^60^ function as essential negative regulators that dephosphorylate MAPK (p38/JNK) to terminate inflammation. Studies indicate that DUSP1 activity is compromised in OA fibroblast-like synoviocytes; notably, its restoration significantly suppresses the expression of MMP13 and COX-2, effectively ‘resetting’ the inflammatory switch. Similarly, DUSP5 has been identified as a specific inhibitor of ERK signaling, protecting chondrocytes from IL-1β-induced catabolism.^61^

Furthermore, protein phosphatase 2 A (PP2A) serves as a broad-spectrum guardian of cartilage homeostasis by restraining the PI3K/AKT/mTOR and NF-κB pathways. However, this reversible mechanism is disrupted in OA by the aberrant up-regulation of CIP2A (cancerous inhibitor of PP2A).^62^ High levels of CIP2A physically complex with PP2A to inhibit its enzymatic activity, thereby preventing the dephosphorylation of key signaling nodes and locking chondrocytes in a sustained pro-inflammatory and senescent state. Thus, therapeutic strategies must evolve to not only inhibit kinases but also reactivate these dormant phosphatase checkpoints.

## Critical limitations and translational challenges

Despite the promising therapeutic targets identified, bridging the gap between bench and bedside requires a critical re-evaluation of experimental inconsistencies and limitations. A primary concern is the chemical promiscuity and dosage of kinase inhibitors used in preclinical studies. Many findings rely on pharmacological probes at high micromolar concentrations (often 10–50 μM) that exceed physiological relevance and compromise selectivity; for instance, the widely used ‘JNK-specific’ inhibitor SP600125 exhibits significant off-target activity against p38 MAPK and other kinases at higher doses, potentially confounding the interpretation of whether observed effects are due to JNK inhibition or broad-spectrum suppression.

Furthermore, the temporal and spatial duality of these signaling pathways poses a therapeutic dilemma. Kinases such as those in the Wnt/β-catenin and NF-κB pathways are not exclusively pathogenic; they are also essential for chondrocyte homeostasis and repair. Consequently, global or sustained inhibition often yields contradictory outcomes, suppressing inflammation while simultaneously impairing cell survival or inducing ossification, which may explain the failure of rigorous pathway blockade in clinical trials.

Finally, the translatability of animal models remains a significant bottleneck. The prevalent use of surgically induced rodent models, such as destabilization of the medial meniscus (DMM), predominantly mimics acute, mechanically driven cartilage loss. These models may not fully recapitulate the slow, chronic metabolic dysregulation and cellular senescence characteristic of human age-related OA, leading to discrepancies in drug efficacy when moving from mice to humans.

## Prospect and conclusion

Protein phosphorylation and dephosphorylation lie at the heart of OA pathogenesis. The phosphorylation status of key signaling pathways like MAPK and NF-κB, profoundly dictates the cellular events that drive disease progression. While significant progress has been made, several areas warrant further investigation to translate this knowledge into effective therapies.

### From linear pathways to dynamic signaling networks

From a reductionist view, it is inadequate to attribute OA pathogenesis to the failure of a single signaling pathway. While pathways such as MAPK and NF-κB are indisputably critical, they do not function as isolated linear cascades but rather as integral components of a highly dynamic and interconnected regulatory network. The signaling output is ultimately determined by extensive and complex crosstalk between these pathways. For instance, the synergy between the MAPK and NF-κB pathways is well-documented; their common upstream activator, TAK1, can be triggered by pro-inflammatory cytokines, leading to parallel activation of both cascades. Downstream, activated MAPKs (particularly p38 and ERK) can significantly enhance the transcriptional activity of NF-κB by phosphorylating and activating kinases like MSK1/2, which in turn can phosphorylate the NF-κB p65 subunit at Ser 276, promoting its interaction with co-activators like CBP/*p*300.^63^^,^^64^ Finally, to bridge the gap between the diverse PTMs introduced at the outset and the phosphorylation-centric focus of this review, it is critical to recognize that these modifications do not operate in isolation. Instead, they engage in extensive hierarchical crosstalk, where phosphorylation often acts as the master regulator. For instance, histone lactylation,^65^ a modification driven by the lactate, is intrinsically linked to the metabolic state of the cell; notably, the glycolytic enzymes that generate this lactate (such as PKM2 and LDHA) are themselves strictly governed by the phosphorylation. Similarly, lysine malonylation has been identified as a key regulator of chondrocyte senescence via Sirt5. Given that sirtuin activity and localization are frequently modulated by AMPK-mediated phosphorylation,^66^ this suggests that a coordinated ‘phosphorylation-acylation’ axis orchestrates joint homeostasis. Therefore, future research should not view these PTMs as separate entities, but rather investigate how the phosphoproteome dictates the landscape of these emerging metabolic modifications.

Furthermore, other critical pathways are deeply intertwined within this network. The PI3K/AKT pathway, a central regulator of cell survival and metabolism, cross-communicates extensively with inflammatory pathways. Activated AKT can modulate NF-κB signaling, often in a complex, context-dependent manner, and its dysregulation is closely linked to chondrocyte apoptosis and senescence in OA.^9^ Importantly, the signaling network is not static but is governed by sophisticated feedback and feed-forward loops that are also controlled by phosphorylation. A classic example is the negative feedback loop where activated MAPKs induce the expression of DUSPs, which then dephosphorylate and inactivate the MAPKs, thus controlling the duration and amplitude of the inflammatory signal.^67^

Therefore, future research must switch from the “linear thinking” of single-pathway analysis to the “network thinking”. This paradigm shift necessitates the integration of high-throughput, multi-omics data, especially quantitative phosphoproteomics combined with transcriptomics and metabolomics, to build comprehensive, predictive models of the chondrocyte signaling network. By applying computational and bioinformatic tools to analyze these complex datasets, researchers can identify critical “nodes”, “hubs”, or “bottlenecks” within the network. These nodes, which integrate signals from multiple pathways, may represent far more effective and robust therapeutic targets than any single component of a linear cascade.^68^

This systems-level understanding redefines the therapeutic goal. A more sophisticated and ultimately more effective strategy may lie not in the aggressive inhibition of a single pathway, but in “network remodeling”, restoring the overall kinase/phosphatase balance within the chondrocyte. Such an approach could involve multi-target, low–dose combination therapies designed to gently guide the cellular state away from a pathological, catabolic/inflammatory phenotype and back towards a physiological, homeostatic, or anabolic one. This strategy of re-establishing signaling homeostasis promises to yield therapies that are not only more effective but also have a superior safety profile compared to potent single-target inhibitors.^69^

### Uncovering the “dark phosphoproteome” with proteomics and artificial intelligence

To bridge the gap between vast phosphoproteomic data and biological insights, specific computational architectures are now being deployed. Recent advancements have utilized Deep Learning, particularly Convolutional Neural Networks (CNNs) and Attention Mechanisms, to predict kinase–substrate relationships. Unlike traditional methods that rely on simple linear motifs, tools such as MusiteDeep and DeepPhos employ multi-layered neural networks to extract high-dimensional features from local amino acid sequences and protein structures, achieving superior prediction accuracy.^70^ Furthermore, network medicine approaches are translating these predictions into therapeutic leads. By mapping dysregulated phosphorylation events onto the human protein–protein interaction (PPI) network, algorithms can identify “disease modules”—clusters of interacting proteins that drive OA pathology. This topological analysis allows for the calculation of “network proximity” between drug targets and OA disease modules, a technique that has successfully identified novel drug repurposing candidates for degenerative diseases.^71^ However, the application of these models in OA is currently constrained by the “sparsity” of cell type-specific training sets (e.g., purified chondrocytes *vs.* whole joint tissue), necessitating the development of few-shot learning algorithms to handle limited data.

The currently identified phosphorylation sites represent only the tip of the iceberg. The functions of thousands of other sites remain unknown. The integration of high-sensitivity, mass spectrometry-based quantitative phosphoproteomics with Artificial Intelligence (AI) and Machine Learning (ML) algorithms offers a powerful approach. These technologies can screen for disease-associated phosphorylation events on a global scale and predict novel kinase–substrate relationships that are undiscoverable by traditional methods.^55^ This vast, unannotated space constitutes a “dark phosphoproteome”, which likely harbors countless undiscovered regulatory mechanisms critical to OA pathogenesis. Omics technologies (such as genomics, transcriptomics, proteomics, metabolomics, and epigenomics) provide comprehensive and multidimensional datasets for gaining a deeper understanding of the molecular mechanisms underlying OA. However, the complexity and high dimensionality of these data make analysis challenging. AI plays a crucial role in omics data analysis. It can integrate multi-omics data and identify key molecular mechanisms and biomarkers, thereby revealing the pathogenesis and progression of OA. Through AI-assisted omics analyses, scientists can gain a deeper understanding of the molecular basis of OA, providing a basis for developing new treatment strategies.^56^ Bridging this knowledge gap requires a technological synergy between high-throughput data generation and sophisticated data analysis. The primary engine for this exploration is mass spectrometry (MS)-based quantitative phosphoproteomics. Modern methodologies, which typically involve the enzymatic digestion of proteins, enrichment of low-abundance phosphopeptides (using techniques like titanium dioxide (TiO_2_) or immobilized metal affinity chromatography (IMAC)), and high-resolution MS analysis, now permit the identification and quantification of over 10,000 unique phosphorylation sites from a single experiment.^57^ By applying quantitative strategies such as SILAC, TMT, or label-free quantification, researchers can create high-resolution maps of signaling dynamics, comparing healthy versus OA cartilage or chondrocytes in response to pathological stimuli like IL-1β. This approach has already begun to reveal widespread, previously unappreciated changes in the chondrocyte phosphoproteome during OA progression.

However, the sheer volume and complexity of the data generated by these experiments present a formidable analytical challenge that necessitates the use of AI and ML. These computational tools are indispensable for extracting meaningful biological insights from the data in several key ways.

### Enhanced kinase-substrate prediction

Traditional methods for assigning kinases to phosphorylation sites often rely on identifying short, linear “consensus motifs” around the site, an approach with limited accuracy. In contrast, modern ML algorithms, particularly deep learning models like convolutional neural networks (CNNs), can be trained on the vast repository of known kinase-substrate pairs. These models learn complex, non-linear patterns, including distal sequence information and structural context, to predict the responsible kinase for a given orphan phosphorylation site with far greater accuracy.^72^

### Predicting functional consequences and network integration

AI can move beyond simple kinase-substrate pairing to predict the functional impact of a specific phosphorylation event. ML models can be trained to predict whether a modification is likely to switch an enzyme on or off, disrupt a protein–protein interaction, or create a docking site for an adapter protein (e.g., a 14-3-3 protein). This allows for the functional annotation of newly discovered sites at scale, enabling their integration into more accurate and predictive models of cellular signaling networks.^73^

### Advancing personalized medicine through patient stratification

OA is a heterogeneous disease, and different subtypes may respond differently to specific therapies.^74^ Identifying biomarkers associated with specific phosphorylation events or pathway activities could help stratify patients for targeted treatments. Personalized medicine approaches could improve the success rate of clinical trials and lead to more effective treatments for individual patients. Tailoring treatments based on individual patient characteristics, including their specific phosphorylation patterns and pathway activities, holds promise for improving OA management. Recognizing OA heterogeneity is crucial for developing effective personalized therapies. OA represents a heterogeneous group of debilitating joint disorders rather than a single disease entity. Its pathogenesis is complex, driven by diverse factors, including age, genetic predisposition, mechanical injury, obesity, and metabolic dysfunction. This inherent heterogeneity contributes significantly to the limited success of broadly applied therapeutic strategies and underscores the critical need for precision medicine approaches tailored to distinct patient subtypes. Targeting dysregulated phosphorylation-dependent signaling pathways with specific kinase inhibitors offers a promising strategy for personalized OA treatment. However, the clinical efficacy of such interventions is likely contingent upon accurate patient stratification and the selective targeting of pathways predominantly active within a specific OA subtype. This discussion explores a rationale for selecting phosphorylation-targeting inhibitors based on OA patient subtypes, focusing particularly on inflammatory and metabolic phenotypes (Table 1).

**Table 1:** Identifying clinically relevant OA subtypes and selection of kinase inhibitor targets.

Patient Subtypes	Clinical Characteristics	Validated Biomarkers	Selection of Kinase Inhibitor Targets
Inflammatory OA phenotype	Prominent synovitis (detectable via MRI or ultrasound)	Serum/synovial fluid cytokine profiles	JAK/STAT pathway
	Joint effusion	CRP levels	NF-κB pathway
	Joint swelling	Quantitative imaging scores for synovitis	MAPK pathways
	High pain intensity	Potentially specific gene expression signatures derived from synovial tissue biopsies	NGF/TrkA pathway
Metabolic OA phenotype	Obesity	Clinical criteria for MetS	PI3K/Akt/mTOR pathway
	Insulin resistance or type 2 diabetes	Serum adipokine levels (leptin/adiponectin ratio)	AMPK pathway
	Hypertension	Markers of glucose homeostasis (glucose, insulin, HbA1c)	MAPK pathways
	Dyslipidemia	Lipid profiles	
		Distinct metabolomic signatures in biofluids	
		Levels of advanced glycation end-products (AGEs)	

ML algorithms excel at identifying subtle patterns in complex datasets. By training models on phosphoproteomic profiles from large patient cohorts, it becomes possible to identify a minimal set of phosphorylation events, a “phospho-signature” that can accurately classify individuals with OA, predict disease progression, or even stratify patients into distinct molecular subtypes (e.g., inflammatory-driven *vs.* metabolic-driven OA). Such signatures hold immense potential as future diagnostic tools or as biomarkers for evaluating therapeutic response.^75^ Additionally, the potential for personalized medicine approaches should be investigated further.

In conclusion, the powerful combination of MS-based phosphoproteomics for global data acquisition and AI/ML for intelligent data interpretation is poised to illuminate the dark phosphoproteome. This integrated approach will dramatically accelerate the discovery of novel regulatory nodes in OA, validate new drug targets, and identify robust biomarkers, heralding a new era of data-driven, systems-level investigation into the disease.

Finally, novel research avenues and technological advancements should be explored. This includes utilizing advanced proteomics and phosphoproteomics techniques for comprehensive analysis of phosphorylation dynamics in OA, employing single-cell sequencing technology to understand phosphorylation patterns in different cell populations within the joint, developing more specific and potent phosphorylation inhibitors with improved pharmacokinetic properties; exploring the application of gene therapy and other novel therapeutic modalities targeting phosphorylation pathways,^74^ investigating small molecules or peptides in the regulation of phosphorylation-related proteins in OA,^76^ and leveraging artificial intelligence and machine learning to identify new phosphorylation targets and predict drug efficacy.^77^ Continuous advancements in research technologies are essential for deepening our understanding of phosphorylation in OA and developing innovative therapies.

### The critical role of phosphatases in OA pathogenesis

While kinases function as signal “writers” that initiate phosphorylation cascades, protein phosphatases act as essential “erasers” or “editors”, actively regulating the duration and amplitude of these signals to maintain chondrocyte homeostasis. The pathogenesis of OA is increasingly viewed not merely as kinase hyperactivation, but also as a failure of this “writing-erasing” equilibrium. A quintessential example is protein phosphatase 2 A (PP2A), a major serine/threonine phosphatase that functions as a critical guardian of cartilage integrity. PP2A exerts a broad protective effect by acting as a molecular “brake” on multiple catabolic networks; it directly dephosphorylates key components of the Wnt/β-catenin and mTOR/AKT pathways, thereby preventing the hypertrophic differentiation and autophagy defects driven by these “writers”. Furthermore, recent mechanistic breakthroughs have revealed that the deubiquitinase MYSM1 recruits PP2A to dephosphorylate the kinase RIPK2, effectively dampening NF-κB-driven synovial inflammation; conversely, the loss of this PP2A-mediated checkpoint exacerbates joint destruction. These findings highlight that restoring the activity or specific recruitment of phosphatases represents a promising strategy to re-establish signaling balance in OA. To date, OA research and drug development have overwhelmingly focused on the inhibition of protein kinases. Protein phosphatases, the equally important counterparts, have been relatively under-explored. Phosphatases, such as DUSPs that dephosphorylate both threonine and tyrosine residues in the MAPK activation loop, serve as essential negative regulators. Enhancing the activity of specific DUSPs could be a novel therapeutic strategy to dampen excessive MAPK signaling.^78^ Beyond the DUSPs, other specific phosphatases function as critical checkpoints in maintaining chondrocyte homeostasis. For instance, the protein tyrosine phosphatase SHP2 (encoded by PTPN11) has been identified as a guardian of cartilage integrity. SHP2 coordinates a homeostatic network that prevents premature chondrocyte hypertrophy and catabolism.^79^ Genetic ablation of Ptpn11 in chondrocytes disrupts this balance, leading to severe joint deformities and accelerated OA progression. These examples underscore that phosphatases are not merely passive “off switches” but active network homeostatic balancers essential for joint health.” A concerted effort to characterize the role of key phosphatases in OA and develop specific modulators (activators or inhibitors) will undoubtedly open new avenues for OA treatment.

In summary, targeting the phosphorylation/dephosphorylation machinery may offer a promising strategy for OA therapy. This approach possesses several key advantages. As a molecular switch, phosphorylation provides a mechanism for the rapid and potent modulation of cellular behavior. Unlike genetic interventions, pharmacological modulation of phosphorylation is reversible, offering a superior safety profile. The ATP-binding pockets and activation loops of many kinases are well-defined, making them highly tractable targets for the rational design of small molecule drugs. A continued and deepened exploration of the phosphorylation regulatory network in OA will not only enhance our fundamental understanding of this debilitating disease but also pave the way for the development of next-generation therapeutics that could bring new hope to millions of patients worldwide.

## CRediT authorship contribution statement

**Jingwen Li:** Writing – review & editing, Writing – original draft, Validation. **Muhammad Umar:** Writing – original draft. **Xin Dai:** Writing – original draft. **Yu-Ching Hsu:** Writing – original draft. **Hongxun Sang:** Validation, Funding acquisition. **Di Chen:** Writing – review & editing, Validation, Funding acquisition. **Ke Lu:** Writing – original draft, Supervision, Investigation, Funding acquisition.

## Funding

This project was supported by the 10.13039/501100012166National Key Research and Development Program of China (No. 2022YFA1207500), 10.13039/501100001809National Natural Science Foundation of China (No. 82302757, 82394445), 10.13039/501100004791Shenzhen Science and Technology Program (China) (No. JCY20240813145204006, JCYJ20250604183541052, SGDX20201103095600002), 10.13039/501100004791Shenzhen Medical Research Funds (China) (No. B2502005, B020602), Shenzhen Development and Reform Program (China) (No. XMHT20220106001), Shenzhen Key Laboratory of Digital Surgery 3D Printing (China) (No. SYSPG20241211173844006), Guangdong Provincial Engineering Technology Research Center for Clinical Translation and Application of Medical 3D Printing Materials (China) (No. 2023B192).

## Conflict of interests

The authors declare that there is no conflict of interests.
